# Current trends and insights on EMS mutagenesis application to studies on plant abiotic stress tolerance and development

**DOI:** 10.3389/fpls.2022.1052569

**Published:** 2023-01-04

**Authors:** Liuzhu Chen, Liu Duan, Minghui Sun, Zhuo Yang, Hongyu Li, Keming Hu, Hong Yang, Li Liu

**Affiliations:** ^1^ State Key Laboratory of Biocatalysis and Enzyme Engineering, Hubei Collaborative Innovation Center for Green Transformation of Bio-Resources, Hubei Key Laboratory of Industrial Biotechnology, School of Life Sciences, Hubei University, Wuhan, China; ^2^ Jiangsu Key Laboratory of Crop Genomics and Molecular Breeding/Key Laboratory of Plant Functional Genomics of the Ministry of Education, College of Agriculture, Yangzhou University, Yangzhou, China; ^3^ Co-Innovation Center for Modern Production Technology of Grain Crops of Jiangsu Province/Key Laboratory of Crop Genetics and Physiology of Jiangsu Province, Yangzhou University, Yangzhou, China

**Keywords:** ethyl methane sulfonate, molecular breeding, abiotic stress tolerance, plant development, mutation breeding

## Abstract

Ethyl methanesulfonate (EMS)-induced mutagenesis is a powerful tool to generate genetic resource for identifying untapped genes and characterizing the function of genes to understand the molecular basis of important agronomic traits. This review focuses on application of contemporary EMS mutagenesis in the field of plant development and abiotic stress tolerance research, with particular focuses on reviewing the mutation types, mutagenesis site, mutagen concentration, mutagenesis duration, the identification and characterization of mutations responsible for altered stress tolerance responses. The application of EMS mutation breeding combined with genetic engineering in the future plant breeding and fundamental research was also discussed. The collective information in this review will provide good insight on how EMS mutagenesis is efficiently applied to improve abiotic stress tolerance of crops with the utilization of Next-generation sequencing (NGS) for mutation identification.

## 1 Overview of EMS mutagenesis

Ethyl methanesulfonate (EMS) is one of the most common used alkylating agents that can induce chemical modification of nucleotides through the introducing active alkyl group, which creates base changes and nucleotide mutations (Sabetta et al., 2011; Arisha et al., 2014; Gillmor and Lukowitz, 2020). Modifications caused by alkylating agents include the N^7^ and O^6^ of guanine (G), N^3^ and N^7^ of adenine (A), N^3^, O^2^, and O^4^ of thymine (T), N^3^, O^2^, and N^4^ of cytosine (C), and the phosphonate backbone (Murray, 1987; Fu et al., 2012). The EMS primarily induces the changes in guanine (Boysen et al., 2009). Compared to cytosine, the O^6^-exhalation pair is more stable than the thymine pair, so during replication the O^6^-GC pair is regularly converted into an AT pair. A lot of studies for EMS in the genotoxic activity *in vivo* and *in vitro* provide clear evidence, that EMS is a carcinogenic chemical and it is necessary to be very careful when using it (Gocke et al., 2009).

In the vast majority of cases, the mutant phenotype is due to premature stop codons, splice site disruption, deleterious amino acid substitutions, and loss of gene function (Sabetta et al., 2011). Therefore, it is critical to ensure that a large quantity of mutant alleles are present in a small mutant population and that the requirements for efficient homogenization are adequately met.

EMS is a type of non-transgenic chemical mutagen, and EMS mutagenesis is an important way to obtain mutations and the discovery of new genes for plants. Special protocols have been established for many plant species (Unan et al., 2021). The first commercial rice varieties CL112 and CL141, which were M_2_ from Clearfield rice varieties AS350, promoted the imidazolinone (IMI) resistant commercial progress and has important significance for rice research (Sudianto et al., 2013). Treatment with EMS is inexpensive, easy to implement, and induces point mutations at high rates and with good agreement in most species with different genetic backgrounds (Gillmor and Lukowitz, 2020). Currently, EMS mutagenesis can achieve a large number of mutants in a short period of time, which can facilitate the study of stress tolerance in plants. Moreover, it is necessary to strengthen research of phenomics, transcriptomics, proteomics, metabolomics, and other methods in EMS mutant screening and gene function analysis. It is significant carrying out research on stress tolerance breeding from different directions, and tap the potential of EMS mutagenesis in abiotic stress tolerance research.

## 2 The application of EMS mutagenesis

When EMS mutates seeds or tissues, several key parameters need to be considered, such as the uniformity of the material to be mutated and the optimization of EMS dose. In a perfect world, mutagenic material should be homologous and isogenic would facilitate evaluation using molecular markers (Sabetta et al., 2011). The use of homogeneous materials reduces the ambiguity between phenotype and genotype. After mutagenizing a plant, the first generation of seeds can be selected and selfed in successive rounds to create a large near-isogenic homozygous seed bank (Till et al., 2003). Homozygote is difficult to achieve in obligate vegetative breeding species, such as bananas (Wang X et al., 2021). However, homogeneous populations with fixed heterozygote and common features in all plants can be produced by clonal reproduction.

The second key parameter and main troubleshooting area in mutagenesis experimentation is optimization of EMS dose. The accumulation of induced mutation densities must be balanced against the viability or fecundity of the material so as to be feasible under existing resource conditions. Unan et al. (2021) believe that rice seed pre-soaking for 12 hours, can effectively promote the mutagenic effect of EMS, and establish the standard of rice EMS mutagenesis. Additionally, the shelf life of EMS is critical. Once the mutagen is turned on, EMS can interact with the outside air, which may reduce its activity.

### 2.1 EMS is applied to plant research

EMS mutagenesis is suitable for most plants (Kim et al., 2006). For crops, vegetables, and fruits, the purpose of mutagenesis is to obtain varieties with excellent traits. By identifying the agronomic and quality traits of progeny, a population of progeny with the visible mutation type is obtained, followed by further gene function research. As another example, improving ornamental plant ornamental value and stress resistance is a primary purpose of mutagenesis research. For example, common ornamental plants such as *Chrysanthemum*, moss, and jasmine were applied mutagenesis to obtain new landscape plant varieties (Hossain et al., 2006).

EMS mutagenesis has been used for multi-omits combined analysis, and related genes are obtained by reverse genetics. Subsequently, gene-editing technology is used for research. Editing and recombining genes are the basis for an ideal breeding method, which will bring infinite possibilities and breakthrough developments for human beings in plant genetics. In recent years, these are the primary methods of forwarding genetics among model plants.

### 2.2 Various plant tissues could be used for mutagenesis

Mutagens can make abundant mutations in different organs of plants, such as seeds, bulbs, callus, pollen, etc. (Mba et al., 2010). Plants make different mutagenesis sites, which can be selected according to the needs of the experiment, and EMS can be used for mutagenesis.

Jankowicz-Cieslak et al. (2012) considered that seeds are the most common mutagenic plant materials, and there is also a wealth of data in other plant species on doses used to achieve high densities of induced mutations. In general, for plants propagated by seeds, their seeds are usually preferentially selected mutagenic material (Gottwald et al., 2009). At present, reports on seed mutations are multifarious, and the materials cover a wide range, including food crops, cash crops, vegetables, fruits, and flowers (Chen et al., 2021). During mutagenesis, seeds are soaked in the mutagenesis solution, and the mutagenesis of plants can be achieved by changing the concentration of EMS and the mutagenesis duration. Mutagenesis of plant seeds is relatively convenient and quick, and not only are the steps simple, the workload is small, and it is suitable for the experimental research that requires the mutagenesis of most plants. Of course, we prefer the method of seed mutagenesis, but some plants may not be able to use the traditional method of seed mutagenesis due to different experimental purposes. It is not easy to obtain homozygous offspring from seeds, and it needs to be screened by techniques such as hybridization. At this time, pollen, callus, spores, plant fragments, etc. can also be selected for mutagenesis, and the mutagenesis method is relatively complicated. Compared with EMS mutagenesis of seeds, EMS mutagenesis of plant pollen has a large workload and complex operation in the process of mutagenesis. A common method is *in vivo* mutagenesis, that is, mutagenesis is carried out in a pollen tube. Meanwhile, other mutagenesis sites are usually selected for *ex vivo* mutagenesis methods.

## 3 The principles, methods and process of EMS mutagenesis

### 3.1 The concentration of the chemical and the duration of mutation

There is a strict correlation between EMS concentration, mutagenesis duration and lethality. Singh and Sadhukhan (2019) used EMS to mutate three diverged genotypes of grasspea, viz *Nirmal*, *Biol-212* and *Berhampur local*. The results showed that when the EMS concentration of *Nirmal* and *Biol-212* was higher than 0.5% and that of *Berhampur local* was higher than 1%, it was found to be fatal (Singh and Sadhukhan, 2019). The most effective concentration of EMS was 0.5%. In addition, with the increase in EMS concentration, the mutagenic efficiency increased and the effectiveness decreased (Singh and Sadhukhan, 2019). Ghosh et al. (2020) treated terminal cuttings with 0.25%, 0.30%, 0.35% and 0.40% of EMS, respectively. They concluded that morphological as well as flowering parameters, namely flower bud length, plant height, leaf area, flower yield and number of primary branches, decreased with increasing mutagenic dose. The study by Roychowdhury and Tah (2011) showed that germination and survival rates were significantly reduced with increasing EMS levels. The tolerance of plants to EMS varies widely, for example, in *Arabidopsis*, a 0.25-0.5% EMS mutation rate is high, tomato farming populations are constructed by 0.7% and 1% EMS, while in cucumber, 1.5-2% EMS (Greene et al., 2003; Minoia et al., 2010). In common plants, the larger the seed, the higher the EMS concentration required, and the longer the mutagenesis duration necessary, in order to achieve a certain mutation effect.

**Figure 1:**
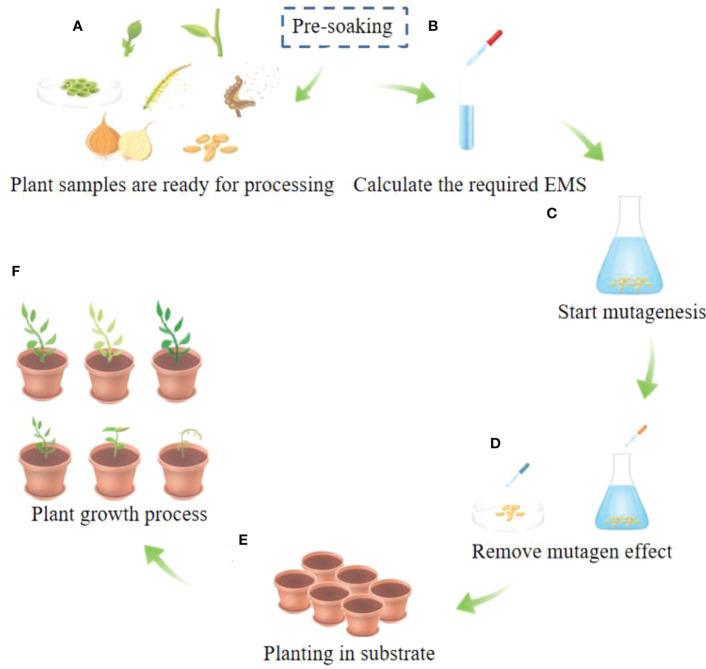
General steps of EMS mutagenesis. **(A)** Different prats of plants can be used for mutagenesis. The parts of the plant shown in A are buds, branches, callus, pollen, spores, bulbs, seeds and other parts of the plant tissue. **(B)** Determine the required EMS mutagenesis concentration and mutagenesis duration to profrom mutagenesis, and perform the mutagenesis. The preferred mutagenesis concentration range is 0.01%-4% and the duration range is 0.5h-12h. **(C)** Start mutagenesis with plant seeds as an example. **(D)** Rinse after mutagenesis or add mutagenesis terminator. **(E)** Planting in culture substrate. **(F)** After the seedlings grow, various phenotype can appear and carry out identification and further experiments.

Mutagenesis duration is also different for different plants. In general, the higher the mutagenesis concentration selected, the shorter the mutagenesis duration. Conversely, the longer the mutagenesis duration, the lower the mutagenesis concentration used. The mutagenesis concentration required for different mutagenesis positions is also different. The mutagenesis concentration of seed mutagenesis and stem node mutagenesis is relatively high, while that of pollen mutagenesis is relatively low. When using plant seeds for mutagenesis, rice was mutagenized with 0.7% EMS, while pepper was mutagenized with 0.02% EMS concentration (Dwinianti et al., 2019; Yan W et al., 2021). The choice of concentration may also be related to the size of the seeds. Mutagenesis may also be affected by the mutagenic environment, which has not been proven due to the uncertain outcome of mutagenesis. When experimenters conduct EMS mutagenesis, they generally refer to published data and make appropriate choices based on the actual situation, instead of blindly conducting experiments according to the published data. In general, 50% of the lethality can be a good mutagenesis effect (Ke et al., 2019). In fact, in order to pursue higher mutation efficiency, the lethality rate we choose generally reaches 80%. Although the specific operation process is different, the basic steps are almost the same, as shown in **Figure 1**. The EMS mutagenesis concentrations of common plants are shown in **Table 1**.

**Table 1:** EMS mutagenesis study in different plants.

Plant species	Mutagenic concentration and duration	Combined with other technologies	Mutagenic significance	Reference
Four *Chrysanthemum* cultivars (‘*Homa’, ‘Fariba2’, ‘Arina’*, and *‘Delkash’*)	0%, 0.125%, 0.25%, and 0.5%	Genetic polymorphism among mutants and their parents was detected using inter simple sequence repeat (ISSR) and inter-retrotransposon amplified polymorphism (IRAP) molecular markers.	A wide range of phenotypic leaf and inflorescence variability was obtained.	Nasri et al., 2022
Marigold (*Tagetes* sp.)	0%, 0.2%, 0.4%, 0.6%, 0.8%, 1.0%, 1.5%, 2.0%, and 3.0%; 4 hours (The acute mutation technique), 6 hours, 24 hours and 48 hours (The chronic mutation technique)	Observations were made on the percentage of surviving plants, quantitative and qualitative characters.	Increasing the diversity of marigold (*Tagetes* sp.).	Lenawaty et al., 2022
Sweet wormwood (*Artemisia annua* L.)	100 Gy, 200 Gy, 300 Gy and combination treatments with 100 Gy + 0.1%EMS, 200 Gy + 0.1% EMS, 300 Gy + 0.1%EMS	Meiotic study was done and various cytological aberrations were observed. In addition, quantitative analysis of chl pigments was also done.	Increasing the genetic variability and induces new trait.	Singh and Kumar, 2022
Four watermelon accessions named *G42*, *97103*, *PI 595203* and *PI 296341-FR*	0%, 0.1%, 0.15% and 0.2%; 0, 40, 60 and 80 min	Incorporating the high-coverage and accurate long-read sequence data.	Elongated fruit shape and male sterility (*ClMS1*).	Deng et al., 2022
Cotton (*Gossypium hirsutum* L.)	–	Correlation coefficients were used to study the association between fiber traits.	One (Population R) focused on improving four fiber attributes (micronaire, length, strength and elongation) and the other (population S) to pyramid superior alleles for fiber length.	Patel et al., 2022
Chinese cabbage (*Brassica rapa ssp. Pekinensis*)	0.4% EMS at room temperature in the dark for 16 h	A total of 300 M_2_ to M_5_ EMS mutants were phenotypically screened and then sequenced. A forward-genetics approach and reverse-genetics approach were used.	Facilitating gene mining of Chinese cabbage and might also be useful for the study of other *Brassica crops*.	Sun et al., 2022
Aerobic rice cultivar *Nagina 22*	–	Molecular genetics approaches	Salinity tolerance	Shankar et al., 2021
Oilseed rape (*Brassica napus*)	–	Genomic background selection combined with marker-assisted selection	Inducing random mutations throughout the genome with high mutation density	Karunarathna et al., 2021
Maize inbred line *Jing 724*	–	Bulk-segregant RNA sequencing and Kompetitive Allele-Specific PCR assays	Identifying genomic loci regulating Genic male sterility (GMS)	Shi et al., 2021
Sunflower variety *BARI Surjamukhi-2*	0.2%, 0.4%, 0.5%, 0.6% EMS	Quadratic regression analysis	Qermination rate, survival rate and early seedling growth rate	Habib et al., 2021
Indica variety *HHZ*	0.7% EMS solution for 12 h	The whole genome resequencing and single nucleotide polymorphic sites (SNPs).	DNA repair	Yan W et al., 2021
Common wheat (*T. aestivum* L.)	0.5% EMS for 12 h	Genomic DNA extraction, PCR amplification, and sequence analyses	Herbicide-resistant	Chen et al., 2021
Groundnut	1.2% and 0.4% EMS	Targeting Induced Local Lesions in Genomes (TILLING) approach	Reducing allergenicity and increasing oleic content	Karaman et al., 2021
Short day Indian onion cultivar *Bhima dark red*	0%, 0.2%, 0.4%, 0.6%, 0.8%, 1.0%, 1.2% EMS	Probit Analysis based on germination percentage	Mutagenic agent significantly reduced seed germination and seedling growth parameters in terms of shoot and root length	Singh et al., 2021
Chickpea (*Cicer arietinum* L.)	4% EMS for 10 h	Genetic transformation of tobacco plants	Providing resistance to IMI herbicides	Galili et al., 2021
Cucumber (*Cucumis sativus* L.)	–	Genetic analysis, MutMap+ and Kompetitive Allele Specific PCR (KASP) genotyping	Reducing lethality during seed germination in cucumber.	Wang C et al., 2021
Chinese cabbage (*Brassica campestris* L. *ssp. Pekinensis*)	–	Genetic analysis	Breeding new varieties exhibiting a wax deficient phenotype	Liu et al., 2021
Barley (*‘Hordeum vulgare’* L.)	Five EMS dosages (0.1%, 0.3%, 0.5%, 0.7% and 0.9%) and five exposure times (0.5 hr, 1 hr, 1.5 hr, 2 hrs and 2.5 hrs)	Data was recorded for percent germination, seedling survival, shoot height, root height, shoot and root biomass.	Selecting fodder barley mutants with high biomass yield	Sharamo et al., 2021
Rice (*Oryza sativa L.*)	3% EMS	Being characterized for phenotypic, biochemical and grain qualities	Inducing early flowering mutants in popular rice variety *Bapatla 2231* (*BPT 2231*)	Gautam et al., 2021
Wheat (*Triticum aestivum* L.)	0.5% EMS	Measuring their impact on coleoptile length, gibberellic acid (GA) sensitivity, and DELLA/GID1 interaction	Increasing wheat yields	Jobson et al., 2021
*Kharif* sorghum (*Sorghum bicolor* L.)	0.1%, 0.2%, 0.3% EMS	Chlorophyll mutation frequency and spectrum	Chlorophyll mutants	Thange et al., 2021
Fenugreek (*Trigonella foenum-graecum* L.)	0.1%, 0.2%, 0.3%, and 0.4% EMS	LSD test	Increaseing grain yield and secondary metabolites	Parchin et al., 2021
Two *Chrysanthemum* cultivars, ‘*Jaguar Pink*’ and ‘*Reagent Pink*’	0.1%, 0.2%, 0.3% 0.4%, 0.5% and 0.6% EMS	Data on the number of survival callus, number of germinating callus, and number of callus with shoots after exposure to different concentrations of EMS were noted.	Obtaining genetic diversity in chrysanthemums	Aisyah et al., 2021
Maize	1% for 2 h	Transcriptomic and metabolic changes	The characterization of flavonoid biosynthesis	Dong et al., 2021
*Ajara ghansal*	EMS 0.8%, SA 0.006%, and gamma rays 200 Gy	Seed germination (%), lethality (%), chromosomal abnormality (%), chlorophyll deficient sector (%), pollen sterility (%), and plant survival were determined	The improvement of non-basmati aromatic landrace *Ajara ghansal*	Desai et al., 2021
The four dwarf wheat mutants, *dm1*, *dm2*, *dm3* and *dm4*	1–1.5% EMS for the duration of 4–8 h	Kyoto Encyclopedia of Genes and Genomes (KEGG) analyses	Great advancements in yield improvement	Xiong et al., 2020
*Jasminum grandiflorum* cv.	0.25%, 0.3%, 0.35% and 0.4% of EMS	Molecular analysis based on ISSR data	Developing new cultivars especially in ornamental crops	Ghosh et al., 2020
*Triticum aestivum* (wheat)	1% EMS	Field trials and principal component analysis, DNA sequencing, Alignment of reads to reference genome, SNP calling	Further increasing salt tolerance	Lethin et al., 2020
*Capsicum annuum* pepper “*Micro-Pep*”	0.3% EMS	Targeting Induced Local Lesions IN Genomes (TILLING)	Genetic diversity in useful traits	Siddiqe MI et al., 2020
The bred new wheat line *KD527*	0.3% EMS for 4 ~ 6 h	Chromosomal location analysis	Lesion-mimic and premature aging	Kong et al., 2020
Seeds of carrot inbred line “*17005*”	0.5% EMS for 6 h	Genetic analysis	Enriching the carrot germplasm	Wu et al., 2020
*Brassica napus*	0.4%, 0.8%, 1.2% and 1.6% EMS	Whole-genome sequencing and GC-FID analysis	Genetic improvement of seed oil content and fatty acid composition of *B. Napus*	Van Zelm et al., 2020
*Hyoscyamus niger*	0.00% 0.01%, 0.02%, 0.03%, 0.04%, 0.05%, 0.06%, 0.70%, 0.08%, 0.09% and 0.1% EMS for 1 hr	PCR analysis, DNase treatment and cDNA synthesis and Quantitative real−time PCR	Increased accumulation of scopolamine and hyoscyamine	He et al., 2018
Sorghum	0.00%, 0.5% and 1.0% EMS	Germination percentage and Emergence percentage	Induce genetic variation in the tested sorghum genotypes	Wanga et al., 2020
Cotton (*G. hirsutum* L.)	0.5%, 1.5%, 2.5% and 3.5% EMS for 3 and 6 h	HRM analysis on DNA base level variation, Genome sequencing and SNP identification	Abundant EMS mutant libraries (approximately 12 000) in allotetraploid cotton were successfully obtained.	Lian et al., 2020
Chili Pepper (*Capsicum frutescens* L.)	0%, 0.01%, 0.02%, 0.04% EMS	SSR molecular marker analysis	Increasing genetic variation in chili pepper plants	Dwinianti et al., 2019
Sesame (*Sesamum indicum* L. *Vartilottama; family: Pedaliacea*e)	0.25%, 0.50% and 1.00% EMS for 2, 4 and 6h durations	Phenotypic variables on which observations were performed included plant height (cm), number of primary and total branches per plant, number of capsules per plant, number of seeds per capsule and per plant and total seed yield per plant (g)	Yield and six yield contributing traits	Saha, 2019
The two upland rice genotypes *Dawk pa-yawm* (white rice) and *Dawk kha 50* (red rice)	EMS at the concentration of 0.5%, 0.75%, 1% and 1.25%	Phenotypic variability among mutants	To create new and diverse characters to a natural population	Awais et al., 2019
*Vernonia* (*Centrapalus pauciflorus (Willd.) H.Rob.*)	0.372% EMS for 1 h and 2 h	Determination of seed oil content and fatty acids	Altering seed oil content and fatty acid compositions in selected *Vernonia* accessions	Hadebe et al., 2019

### 3.2 Screening and analysis of the mutation lines

Chemical mutagenesis of seeds results in a long period of duration before full display. In experiments, identification of a population of plant mutants suitable for genotypic or phenotypic screening requires at least the production of M_2_ or later progeny. The duration required to generate a suitable mutagenize population depends on the choice of mutagenic material, and addressing the issue of heterozygote and homozygote is critical.

Plant materials must be non-chimeric before phenotypic or genotype screening, and the duration required varies depending on the species and tissue culture method used. While chimeras may disintegrate in meristem cells within a month of treatment, one or more rounds of post-EMS meristem dissociation and cutting may be required to ensure that all tissues in the resulting plant are genetically homogeneous (Jankowicz-Cieslak et al., 2012). In recent years, the EMS mutagenesis of some plants is shown in **Table 1**.

Mutagenesis using physical mutagens has been used to generate mutant populations in various plants (Stadler, 1929; Chawade et al., 2010). However, gamma irradiation and high-speed neutrons can lead to greater DNA inversion and deletion, which hinders the recognition of genes under the mutant phenotype (Espina et al., 2018). As an alternative, EMS is a chemical reagent commonly used to induce seed mutations. EMS induces high-frequency random point mutations, some of which can produce new termination codons in genes of interest (Jankowicz-Cieslak and Till, 2015).

EMS has been successfully applied to *Solanaceae*, resulting in morphological diversity that promotes improvements in fruit quality, yield, and desirable traits such as male sterility and disease resistance. The expected result of EMS treatment of seeds or vegetative tissues is that plant populations contain high-density induced point mutations. Due to the existence of homologous sequences, the density of polyploid plants is higher. As toddlers first observe phenotype, increased ploidy confers greater tolerance to mutations (Stadler, 1929). The mutation density of triploid banana is 1/57 KB, that of tetraploid wheat is 1/40 KB, and that of hexaploid wheat and oats is 1/24 KB (Slade et al., 2005; Chawade et al., 2010; Jankowicz-Cieslak et al., 2012). The evaluation of tillering mutants density usually involves screening a small number of target gene mutations in hundreds of plants. Alternatively, less representative genome sequencing of the full genome or fewer plants can be used to estimate mutation density (Henry et al., 2014). Stable phenotypic variation should be observed in the first non-chimeric generation (M_2_ for seed mutation) and can be used to determine whether the population is sufficiently mutated (Haughn and Somerville, 1987; Caldwell et al., 2004; Sabetta et al., 2011). The fertility of mutant populations should be reduced in early generations (Gottwald et al., 2009).

The traits mutated by EMS mainly include yield, quality, and resistance (Wang H et al., 2016). EMS has been successfully applied to a large number of plants to produce morphological diversity and promote the improvement of ideal traits (Lethin et al., 2020). Based on the mutation of agronomic and yield traits, new materials with excellent quality can be screened from the mutated offspring after multiple generations of screening and identification.

When a laboratory technician treated saffron with colchicine and EMS together, *ALDH*, *BGL*, and *CCD2* gene expression increased by 2-fold (Samadi et al., 2022). Mutations of beneficial characteristics for wheat include dwarfing, early maturity, large grain, and more tillers (Ferrie et al., 2020). In the report of Kuraparthy et al. (2007) single tillering mutants were screened from the diploid wheat EMS mutant library by molecular technology, and the exact location of tiller-related genes on the chromosome was determined. Related experimenters identified a new type of rice precocious leaf 85 (*psl85*) mutant in the rice mutant population, showing an obvious premature senescence leaf phenotype and dwarfism, and the expression of senescence-related genes was up-regulated. Furthermore, genetic analysis showed that the senescent leaf phenotype is controlled by a single recessive nuclear gene, and further studies on senescence genes in rice were conducted (He et al., 2018). Yan W et al. (2021) randomly induced G/C to A/T conversion in rice genome by EMS. Whole-genome resequencing of 52 rice EMS mutants to investigate potential EMS mutational bias and its possible correlation with sequence background and chromatin structure.

After EMS mutagenesis treatment, experimenters observed a significant increase in the average number of shoots and roots of Hyoscyamus, and the EMS treatment also increased the accumulation of scopolamine and scopolamine in the explants compared to control (Shah et al., 2020).

After EMS, sodium azide (NaN_3_) or gamma-rays mutagenesis, chlorophyll-deficient mutants were most efficiently generated in EMS mutagenesis, three mutants were obtained from soybean seeds, and the progeny isolation of non-nodule mutant plants was completed (Carroll et al., 1986). The experimenter used EMS to mutagenize wheat cv. *Gao 8901*, and isolated the waxy mutant (*Wx-null*) by screening the progeny seeds with KI-I, and there was no significant difference in seed morphology and size compared with the wild type, but the mutants contained more B-type starch granules, while the amylose content was reduced (Pang et al., 2010). In the rape study, the Korean rape variety *Tamla* was used for EMS mutagenesis. The screened mutants were slightly smaller than the control in the early vegetative growth stage, but the oleic acid content in the mutants increased by about 7%. The mutants will be used for developed high oleic rapeseed varieties and conduct related research (Lee et al., 2018). Experimenters performed EMS mutagenesis on 5500 *Gossypium herbaceum* seeds, and about 5% of the progeny showed visible phenotype, such as leaf shape changes and sterility. The morphological and phenotypic evaluation of more than 4,000 plant materials of progeny was carried out, and the results showed significant differences (Kumar et al., 2022). Munda et al. (2022) treated seeds with different doses of EMS, induced morphological mutants of citronella, analyzed the morphological and physiological characteristics of the obtained mutants, increased herbage yield and citronellal content, and increased economic value. In the EMS mutagenesis of *Brassica napus* plants, a dwarf mutant *bnd2* was successfully isolated. Compared with the wild type, the mutant has a shorter hypocotyl and a reduced plant height, and verified that *bnd2* is a single-site recessive mutation that can be used research on the genetic mechanism of Brassica napus (Li et al., 2021). According to official data released by the FAO/IAEA project, 16 pepper varieties have been bred so far through mutation techniques (http://mvd.iaea.org). In pepper mutagenesis, several studies have used high-quality mutant populations obtained from EMS mutagenesis (Arisha et al., 2014). In particular, the EMS mutant population of sweet pepper variety *‘Maoer’* was established to study the genes regulating flower and plant structure (Siddiqe MI et al., 2020).

Nasri et al. (2022) mutated four *Chrysanthemum* varieties (“*Homa*”, “*Fariba2*”, “*Arina*”, and “*Delkash*”) with EMS (0-0.5%) as mutagens and leaf discs as explants to obtain new varieties. In addition, ISSR and IRAP molecular markers were used to detect the genetic polymorphism between the mutant and its parents. The ISSR and IRAP primers used can classify *Chrysanthemum* mutants according to varieties and some extent according to the EMS concentration used, to confirm their effectiveness in distinguishing real mutants, and to allow them to select in advance and reduce the size of the mutant population. In *in vitro* mutagenesis, EMS-induced mutations may be a useful tool to aid in breeding programs for new generation *Chrysanthemum* varieties.

## 4 Recent studies in plants using EMS mutagenesis

Plants are sessile, and they have to withstand various stress tolerance, which largely limit the way plants can survive, so it is crucial to improve their tolerance to stress tolerance. At present, research to screen resistant mutants by mutagenesis has attracted much attention, and stress-tolerant mutants have been successfully screened in many plants (Ge et al., 2018). Improving plant stress tolerance is a powerful measure to improve plant yield. Moreover, there are many studies on improving resistance through EMS mutagenesis (Thangwana et al., 2021; Li et al., 2022).

Salt tolerance has a great impact on plant yield traits, especially the genetic basis of salt tolerance (Costa and Farrant, 2019). However, in *Arabidopsis*, salt tolerant mutants caused by monocyte mutation have been previously reported, revealing the complexity of the genetic basis of plant salt tolerance. The results show that osmotic pressure, osmotic protectant, free radical detoxification, ion transport system, hormone level changes, and hormone-guided communication are all related to salt stress, which further affects plant yield traits (Quesada et al., 2000; Zhao et al., 2021). EMS induction of 275 mutants has been used in different levels of salinity response study, and research shows *N22-L-1013*, *N22-L-806*, and *N22-L-1010* were identified as typical Na excluders (Shankar et al., 2021). After four generations of choice, and in the field experiment carried out, the EMS wheat mutants, showed good salt-tolerant phenotypes (Lethin et al., 2020). *Hitomebore Salt Tolerant 1* (*hst1*) of rice mutants from the 6000 EMS by Takagi et al. (2015) in the mutant screening, and researchers verified the *OsRR22* role in the process response to salt stress. Due to the complexity of plant salt-tolerant and limited experimental data from previous studies, the NaCl tolerance mechanism of the mutants needs to be further investigated. Salt-tolerant crops play an important role in the use of saline-alkali land for agricultural production (Van Zelm et al., 2020).

EMS-induced yellow seeded *Brassica napus* was used to analyze the drought tolerance of mutants at the bud stage and seedling stage, and successfully screened for mutants with strong drought resistance (Tang et al., 2020). Screening in mutant nightshade populations restored alleles with improved content comprised of phenolic compounds, namely anthocyanin and chlorogenic acid (CGA) (Xi-Ou et al., 2017). In this study, the author performed High Performance Liquid Chromatography (HPLC) to determine the concentration of the anthocyanin, and the results showed that the CGA content of mutant was significantly higher than that of WT. RT-PCR anlysis showed that levels of expression of anthocyanin biosynthetic genes were increased in *S9-1*, with the exception of *SmPAL* (Xi-Ou et al., 2017). After EMS mutagenesis, glyphosate-resistant mutant lines were selected from the M_4_ generation of soybean seeds with excellent agronomic characteristics. Wheat and *Chickpea cicer arietinum* L. Plants. beans also won the IMI mutants in this way (Chen et al., 2021; Galili et al., 2021).

In the process of mutant screening, some physiological indexes can be used as stress resistance indexes for resistance screening under different stress conditions (Friesen and Wall, 1986; Sebastian and Chaleff, 1987). Li et al. (2019) induced radish mutation with EMS, which is suitable for obtaining the target characteristics of new radish materials. *DY13* radish seeds were treated with 0.5% EMS to produce sterile mutants. Morphological observation showed that the anthers of these male sterile lines were curled, browned and wrinkled, and their disease resistance and drought tolerance were enhanced. The male sterile line was successfully selected from *DY13* radish, which provides a new and effective method for the selection of the radish male sterile line.

Munda et al. (2022) used an EMS chemical mutagen to induce the morphological mutant of citronella Indus. The mutant has high puncture strength and an enhanced anti-penetration ability due to a curved stem, which further proves the reliability of practical physiological index analysis of stress-resistant mutants. A large number of studies have shown that it is very reliable to screen the resistance of mutated plants according to physiological indexes, and this will speed up the process of plant stress resistance breeding.

In recent years, various attempts have been made to cultivate crops that are tolerant to herbicide doses, which are usually fatal to weeds (Prakash et al., 2020). Different scientific methods have been used to genetically modify crops to cultivate herbicide-tolerant/herbicide-resistant crops (Halford and Shewry, 2000). Herbicide-resistant mutant plants have been found in many crops, such as corn, wheat, cotton, sorghum, sunflower, lentil, and soybean (Anderson and Georgeson, 1989; Newhouse et al., 1991; Rajasekaran et al., 1996). Rizwan et al. (2017) developed an imidazolinone-resistant lentil mutant (*RH44*) through EMS mutation. In general, it is not easy to develop crops with high-dose herbicide resistance. Such herbicides can kill all types of weeds at one duration. However, to improve the resistance of crops to higher herbicide doses, new strategies are being adopted, such as large-scale screening of mutagenic populations, re-mutation of independently isolated mutations, screening, and gene polymerization, and selecting hypothetical herbicide-resistant mutants in different crops (Piquerez et al., 2014).

Among various mutagenic methods, chemical reagents that can produce genetic variation in crops have been widely popular for many years. A mutation strategy is considered to be an important step for producing new and valuable genetic variations (Wei et al., 2022. Many important crops, such as wheat, sesame, rice, grapefruit, cotton, and bananas, have produced mutant-derived varieties. In tomato, EMS-based mutations have been used to generate specific alleles such as a purple leaf color, smaller leaves, and early fruit (Silué et al., 2013). Through EMS mutagenesis, researchers have cultivated Targeting Induced Local Lesions IN Genomes (TILLING) populations containing a mutant phenotype of various quality traits in cucumber and pumpkin (Kami et al., 2000; Prakash et al., 2020). Some M_1_ plants showed chimerism caused by a recessive gene mutation. Some M_1_ plants showed variation in flower organs, delayed flowering and a pale leaf color at maturity. Fruit color and shape also changed in M_1_ plants, consistent with EMS-induced changes in tomato fruit (Gauffier et al., 2016). Because EMS can cause morphological variation in fruit, we believe that EMS mutation may have potential value in fruit quality (Bhat et al., 2017).

## 5 Molecular technology promotes application of EMS mutagenesis technology

In recent years, research on EMS mutagenesis has continued to develop, and the mutagenesis of some plants is shown in **Table 1**. EMS mutagenesis has been widely studied in mutant screening and mutant library construction. At present, some research progress has been made in *Arabidopsis*, rice, soybean, and other plants (DeMarini, 2020). Identifying mutants by plant phenotype is the most direct method, but this has significant limitations, and it is easy to miss important mutations. In addition, some researchers have used molecular markers to analyze the EMS mutants of wheat, and to screen mutants with different glutenin subunit deletions from a wheat EMS mutant library (Grover and Sharma, 2016). With the rapid development of molecular sequencing technology, research is also intensifying to identify mutants with TILLING technology (Manzanares et al., 2016; Taheri et al., 2017). Reverse genetics is a potentially important approach to identify new mutations in genes of interest. Reverse genetics can be applied to plant tillering, regardless of the level of plant genome structure (Kurowska et al., 2011). In contrast to other reverse genetics methods (such as RNAi technology) and T-DNA insertional mutagens, this non-transgenic method does not require transformation (Krysan et al., 1999; Travella et al., 2006). TILLING aims to find nucleotide changes caused by chemical mutagenesis in target genes, to enable changes in protein function (Till et al., 2004). The development of molecular technology will be beneficial to the identification of EMS mutagenesis mutants and promote the application of EMS mutagenesis technology.

EMS is the mutagen of choice, which induces single-nucleotide variation through the titillation of a specific nucleoside, resulting in a broad spectrum of mutations (Henikoff et al., 2004). This may be silencing, nonsense, missed, and splicing mutations in the gene coding region (Boualem et al., 2014). TILLING has been applied to different plants, such as Archbishops thali, rapeseed, soybean, rice, wheat, tomato, sunflower, and tobacco, indicating that this method is an important alternative method for plant species function analysis (Till et al., 2007; Reddy et al., 2012; Bovina et al., 2014; Yan Z et al., 2021). The mutant population induced by EMS was used to target induced local damage (TILLING) in the genome. Further screening and identification of EMS mutants were identified by the combination of morphological analysis and biotechnology, which will facilitate the screening of abiotic stress tolerance mutants, and the preliminary screening of mutants in a more efficient and targeted manner.

## 6 Conclusion

The development of EMS mutagenesis promoted the rapid development of plant functional genomics, resulting in the birth of map-based cloning technology and directed induced local mutation technology (Kim et al., 2006). The latter one effectively combines traditional chemical mutagenesis with mutant screening skills. EMS mutagenesis cause high throughput mutation with low cost, which tremendously speeds up the research for plant genomics and promotes the development of molecular breeding for abiotic stress tolerance plants.

Similar to other breeding methods, EMS mutation also has limitations, including a high level of randomness, low mutation efficiency, and few beneficial mutations. Additionally, it is still difficult to identity, clone and characterize mutations, resulting in limited application of this method in plant species with complicated or polyploid genomes. Although the latest gene editing technology, such as the CRISPR/Cas9 system, which is faster, more accurate, and is widely used in plant abiotic stress tolerance research by altering individual gene loci, it is difficult for genetic improvement of traits controlled by multiple sites. Moreover, the CRISPR/Cas9 system is difficult to apply to the species with little genomic information. On the contrary, EMS mutagenesis results mutation at multi-sites and can obtain abundant traits, which is difficult to be replaced by other mutagenesis breeding. It is also noteworthy that although the CRISPR/Cas9 technology has been widely used in gene editing, EMS mutagenesis is a non-GMO application, which could meet the strict laws for genetic modification of genes.

## Author contributions

LC prepared an outline, first draft. LD, MS, ZY, KH, HL, HY, and LL provided literature support, and prepared the figure and table. HY and LL are corresponding to overall editing and finalization of paper drafts. All authors read and approved the published version of the manuscript.
